# High-fidelity nano-FTIR spectroscopy by on-pixel normalization of signal harmonics

**DOI:** 10.1515/nanoph-2021-0565

**Published:** 2021-12-13

**Authors:** Lars Mester, Alexander A. Govyadinov, Rainer Hillenbrand

**Affiliations:** CIC nanoGUNE BRTA, Donostia-San Sebastian 20018, Spain; attocube systems AG, Eglfinger Weg 2, 85540 Munich-Haar, Germany; CIC nanoGUNE BRTA and Department of Electricity and Electronics, EHU/UPV,Donostia-San Sebastian 20018, Spain; IKERBASQUE, Basque Foundation for Science, Bilbao 48011, Spain

**Keywords:** far-field reflection, infrared spectroscopy, nano-FTIR, nanoscale materials, s-SNOM

## Abstract

Scattering-type scanning near-field optical microscopy (s-SNOM) and Fourier transform infrared nanospectroscopy (nano-FTIR) are emerging tools for physical and chemical nanocharacterization of organic and inorganic composite materials. Being based on (*i*) diffraction-limited illumination of a scanning probe tip for nanofocusing of light and (*ii*) recording of the tip-scattered radiation, the efficient suppression of background scattering has been critical for their success. Here, we show that indirect tip illumination via far-field reflection and scattering at the sample can produce s-SNOM and nano-FTIR signals of materials that are not present at the tip position – despite full background suppression. Although these artefacts occur primarily on or near large sample structures, their understanding and recognition are of utmost importance to ensure correct interpretation of images and spectra. Detailed experimental and theoretical results show how such artefacts can be identified and eliminated by a simple signal normalization step, thus critically strengthening the analytical capabilities of s-SNOM and nano-FTIR spectroscopy.

## 1 Introduction

In scattering-type scanning near-field optical microscopy (s-SNOM) [1], [2], [3], [4] and Fourier transform infrared nanospectroscopy (nano-FTIR) [5], [6], [7], the metalized tip of an atomic force microscope (AFM) is illuminated by a focused laser beam. The tip acts as an optical antenna [8, 9], effectively focusing the incident light into highly concentrated and localized electric near fields at the tip apex. Near-field interaction between the tip and sample modifies the tip-scattered light (referred in the following as to near-field scattering yielding near-field signals). Spatially and spectrally resolved interferometric recording of the tip-scattered light thus allows for nanoscale-resolved mapping at visible, infrared and terahertz frequencies of, for example, the chemical identity of organic and inorganic materials [5], [6], [7, 10], [11], [12], [13], [14], [15], [16], [17], [18], strain [19, 20], metal–insulator transitions [21], [22], [23], [24], [25], carrier concentrations in semiconductors [26], [27], [28], [29], [30], [31], proteins and their secondary structure [32, 33], phase coexistence in organic materials [34], ferroelectric phases [35], or catalytic reactions [36]. The achieved spatial resolution is on the scale of the tip apex radius (typically 25 nm), independent of the illumination wavelength *λ* [37].

In order to obtain pure and thus reliable and quantitative near-field signals, it is key to suppress various background signals. Additive background signals (for example caused by light scattering at the tip-shaft or the sample) are suppressed by oscillating the tip vertically at frequency Ω and recording the detector signal at a higher harmonic *n* of the oscillation frequency, nΩ [38], [39], [40]. Multiplicative background signals (caused by the interference of near-field and background scattering) are removed by combining higher-harmonic demodulation with interferometric detection of the tip-scattered light [1, 3] yielding pure near-field optical contrasts [41] that are determined essentially by the sample’s electrostatic reflection coefficient β=(ϵ−1)/(ϵ+1) , where ϵ is the sample permittivity.

Interestingly, s-SNOM and nano-FTIR can also probe local electric fields near the sample surface, which is frequently exploited, for example, to map the electric fields of infrared antenna structures [42], [43], [44] and to map the electric fields of surface- and volume-confined phonon-polaritons in polar crystals and polar van der Waals materials [45], [46], [47], [48], [49], [50], [51], respectively, and plasmon polaritons on (semi-)conductors and graphene [52], [53], [54], [55], [56], [57]. The polaritons may be launched or reflected at material boundaries that are located up to several tens of micrometers away from the probing tip [45], showing clearly that s-SNOM and nano-FTIR signals originating from local material properties can be masked by electromagnetic fields that are generated far away from the tip – even when additive and multiplicate background signals are fully suppressed. Importantly, the local electric field at the tip apex can also be modified by far-field reflection and scattering of the incident field at a sample surface [58, 59], which is often not considered in s-SNOM mapping and nano-FTIR spectroscopy of dielectric samples. To remove the influence of far-field reflectivity in s-SNOM, McLeod et al. recently showed and analyzed s-SNOM images of near-field signal amplitudes recorded at different demodulation orders *n* [60]. However, the influence of far-field reflections in s-SNOM has neither been studied nor documented systematically so far. Further, it is unclear to what extent the far-field effects can be suppressed and how the signal ratios have to be interpreted.

Here, we provide a detailed analysis of s-SNOM and nano-FTIR data obtained on various representative samples (layers of Au and h-BN on Si substrates, as well as a tobacco mosaic virus on an inhomogeneous substrate), highlighting and documenting that a careful assessment of far-field reflection effects is of critical importance for the correct interpretation of near-field signals. Our results show that far-field reflection and scattering at the sample surface can lead to dramatic qualitative and quantitative modification of near-field amplitude and phase signals even when additive and multiplicative background are fully suppressed. For example, local s-SNOM and nano-FTIR data can exhibit (spectral) signatures from large sample structures that are several micrometers away from the tip. Most importantly, we confirm that these artefacts induced by the sample’s far-field reflection and scattering can be eliminated by calculating and analyzing at each sample position (pixel) the ratio of the complex-valued near-field data (images or spectra) of the *m*th and *n*th demodulation order.

## 2 Results

### 2.1 Basic modeling of near-field scattering in s-SNOM and nano-FTIR

**Figure 1:**
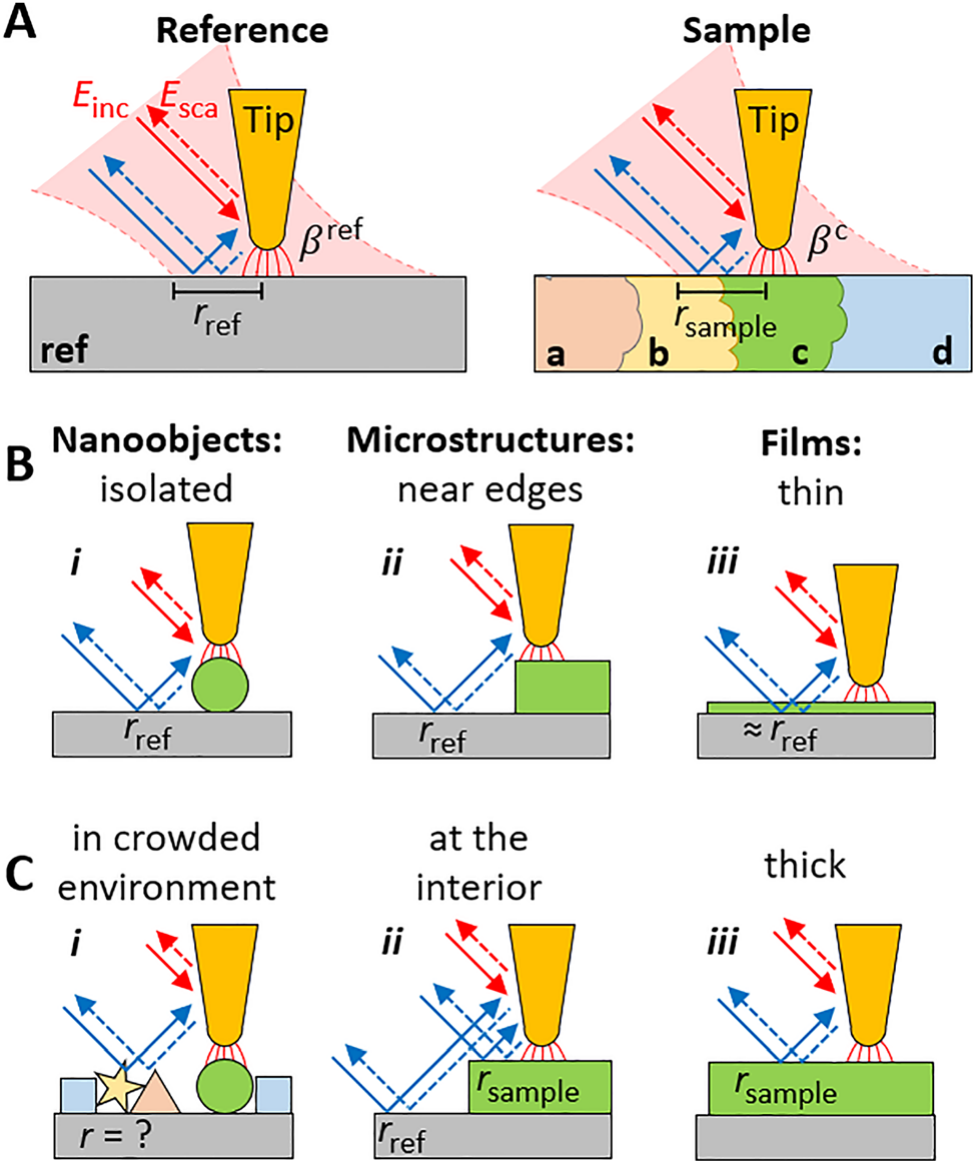
Illustration of typical s-SNOM and nano-FTIR experiments. (A) A metallized AFM tip near a known reference material (left) or unknown sample (right, generic sample composed of materials a, b, c, d) is illuminated by the electric field $E_{\text{inc}}$ of a diffraction-limited infrared laser beam (red area). Near-field interaction between tip and sample modifies the tip-scattered light, $E_{\text{sca}}$ , depending on the electrostatic reflection coefficient $\beta^{j}$ of the material *j* below the tip apex. The tip is illuminated (and the tip-scattered light is detected) directly (red arrows) and indirectly via far-field reflection at the sample surface (blue arrows), depending on the far-field reflection coefficient $r_{j}$ . (B) Situations where far-field reflections cancel upon signal normalization: (*i*) isolated nanoobjects, (*ii*) near edges of extended microstructures, (*iii*) thin films with negligible absorption and reflection, all placed on the reference material. (C) Situations where far-field reflections do not cancel: (*i*) nanoobjects in crowded environments, (*ii*) at the interior of microstructures, (*iii*) thick films, all placed on the reference material and exhibiting significant absorption or reflection.

The tip-scattered field $E_{\text{sca}}$ in s-SNOM and nano-FTIR can be well modeled by [4, 61],

(1) $$E_{\text{sca}}=\sigma E_{\text{inc}}\propto {\left(1+cr\right)}^{2}\alpha_{\text{eff}}\left(\beta \text{, }z\right)E_{\text{inc}}$$

where $\sigma =s{\text{e}}^{\text{i}\varphi }$ is a complex-valued scattering coefficient and $E_{\text{inc}}$ is the incident field provided by an external light source (illustrated in Figure 1A). The near-field interaction between tip and sample is described by the effective tip polarizability $\alpha_{\text{eff}}$ , which depends on the tip-sample distance *z* and the local near-field reflection coefficient β=(ϵ−1)/(ϵ+1) , with ϵ being the dielectric function of the sample below the tip. Equation (1) also accounts for illumination of the tip by the incident field $E_{\text{inc}}$ both directly and via far-field reflection at the sample surface (described by the complex-valued far-field Fresnel reflection coefficient *r* and a factor *c* that describes the weight of the reflected beam compared to the direct illumination, illustrated by blue arrows in Figure 1A). Likewise, the tip-scattered field is scattered towards the detector directly and via reflection at the sample surface (accounted for by squaring the factor 1 + *cr*). Considering interferometric detection and signal demodulation at the *n*th harmonic, the amplitude $s_{n}$ and phase $\varphi_{n}$ signals measured in s-SNOM and nano-FTIR can be expressed by [61]

(2) $$\sigma_{n}=s_{n}{\text{e}}^{\text{i}\varphi_{n}}\propto {\left(1+cr\right)}^{2}\alpha_{\text{eff},n}\text{,}$$

where $\alpha_{\text{eff},n}$ is the *n*th Fourier coefficient of the time-dependent effective polarizability $\alpha_{\text{eff}}\left(\beta \text{, }z\left(t\right)\right)$ with z(t)=A(1+sin(Ωt)) , *z* being the tip-sample distance, *t* the time and *A* the oscillation amplitude (tapping amplitude) of the tip. Clearly, the demodulated amplitude and phase signals, $s_{n}$ and $\varphi_{n}\text{,}$ depend on the far-field reflection coefficient *r* at the sample surface [58, 59]. Further, s-SNOM and nano-FTIR data are typically normalized to a reference measurement (analogous to standard FTIR spectroscopy to eliminate the instrument response function) on a well-known and spectrally flat sample (such as Au or Si), which can be expressed as

(3) $$\sigma_{n}=s_{n}{\text{e}}^{\text{i}\varphi_{n}}=\frac{\sigma_{n}^{\text{sample}}}{\sigma_{n}^{\text{ref}}}=\frac{{\left[1+cr_{\text{sample}}\right]}^{2}\alpha_{\text{eff,}n}^{\text{sample}}}{{\left[1+cr_{\text{ref}}\right]}^{2}\alpha_{\text{eff,}n}^{\text{ref}}}\text{.}$$

In Figure 1A, we sketch a typical reference and a general sample measurement, illustrating that according to Eq. (3), the demodulated (background-free) normalized near-field signal $\sigma_{n}$ on sample area *c* is a mixture of $\alpha_{\text{eff,}n}^{c}\left(\beta^{c}\right)$ and an average far-field reflection coefficient $r_{\text{sample}}$ that is determined by various sample areas (here *c* and *b*). Hence, the normalized near-field signal cannot be attributed exclusively to the near-field-probed area below the tip apex (i.e., to area *c*). However, on many samples the far-field terms ${\left(1+cr\right)}^{2}$ for sample and reference measurements cancel because of $r_{\text{sample}}$ being identical or nearly identical to $r_{\text{ref}}$ [62], yielding near-field signals that exclusively stem from the near-field interaction, $\sigma_{n}\propto \alpha_{\text{eff,}n}^{\text{sample}}$ . We highlight three typical cases in Figure 1B, illustrating the near-field probing of (*i*) an isolated nanoscale object on a substrate that corresponds to the reference surface [12, 30, 33], (*ii*) a sample area in close proximity to the reference area such that far-field reflection dominantly occurs at the reference surface, and (*iii*) a layer on the reference surface, which is thin enough such that its far-field absorption and reflection is negligible [15, 62]. On the other hand, the far-field term ${\left(1+cr\right)}^{2}$ may not cancel (Figure 1C) when studying (*i*) nanoparticles in crowded environments, (*ii*) the interior of micrometer-scale sample areas [29], and (*iii*) thick layers on the reference surface such that their absorption and reflection is not negligible. In the following, we thus study how non-negligible far-field reflection at the sample surface may influence s-SNOM and nano-FTIR results.

### 2.2 Demonstration and elimination of far-field reflection artefacts in s-SNOM

**Figure 2:**
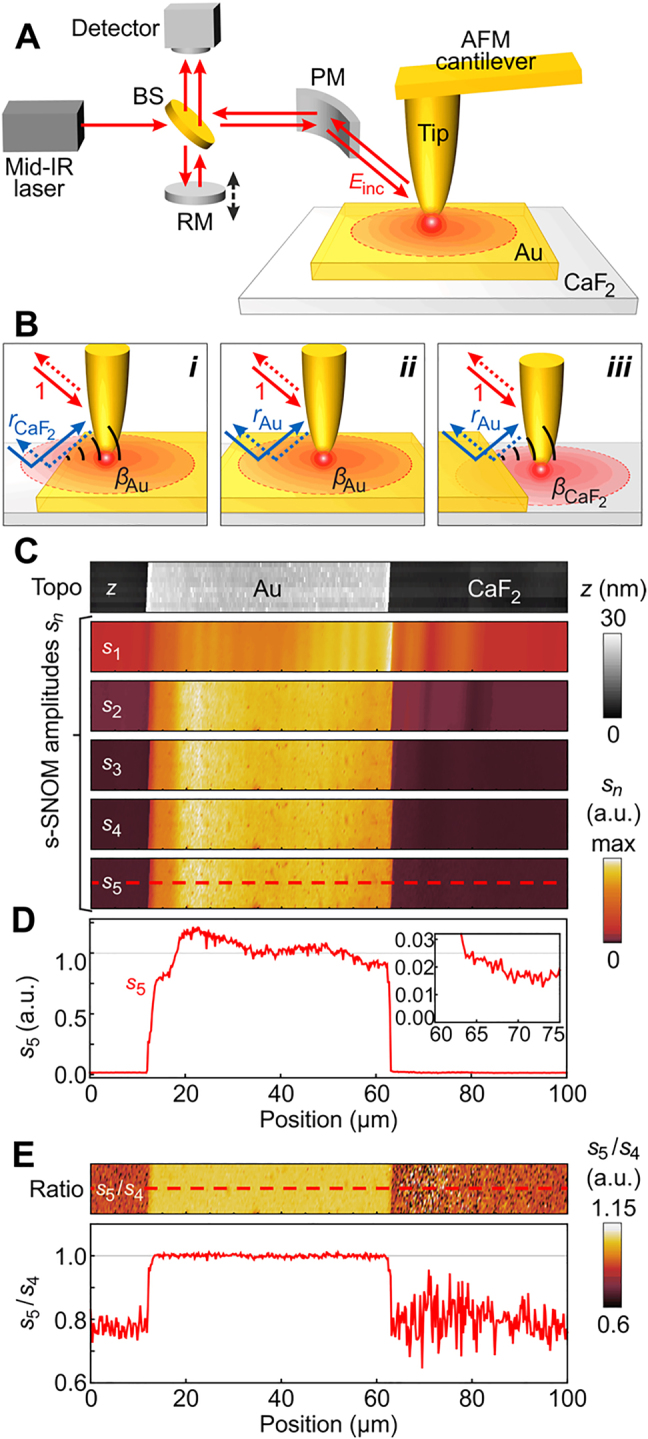
Experimental demonstration and elimination of far-field reflection artefacts in s-SNOM imaging. (A) Experimental setup. The electric field $E_{\text{inc}}$ generated by a single-frequency (for s-SNOM imaging) or broadband (for nano-FTIR spectroscopy) mid-IR laser source is focused with a parabolic mirror (PM) onto an AFM tip that is in intermittent contact (tapping mode) with the sample (here a 25 nm-high Au square on ${\text{CaF}}_{2}$ substrate). The tip-scattered light is collected by the same PM and recombined via a beam splitter (BS) with a beam coming from a movable reference mirror (RM) at the detector position (Michelson interferometer). (B) Illustration of three different probing scenarios, described by: (*i*) $\sigma_{n}\propto {\left(1+cr_{{\text{CaF}}_{2}}\right)}^{2}\alpha_{\text{eff},n}^{\text{Au}}$ , (*ii*) $\sigma_{n}\propto {\left(1+cr_{\text{Au}}\right)}^{2}\alpha_{\text{eff},n}^{\text{Au}}$ , and (*iii*) $\sigma_{n}\propto {\left(1+cr_{\text{Au}}\right)}^{2}\alpha_{\text{eff},n}^{{\text{CaF}}_{2}}$ . Black arcs illustrate light scattered at the Au edge. (C) s-SNOM topography and amplitude $s_{n}$ images recorded at demodulation orders *n* = {1 … 5}, with tapping amplitude *A* = 75 nm, tip radius *R* = 30 nm (Au tip, Nanosensors PPP-NCSTAu) and illumination wavenumber $\nu =1670{\text{cm}}^{-1}$ . (D) Line profile along the dashed line in panel (C). Inset: zoom-in. (E) Image and line profile showing the amplitude ratio $s_{5}/s_{4}$ .

For a most basic example demonstration of far-field reflection artefacts, we performed s-SNOM imaging of an Au patch on CaF_2_. We employed a commercial s-SNOM (neaSNOM from Neaspec GmbH) comprising pseudo-heterodyne interferometric detection and *n*th order higher harmonic demodulation (Figure 2A). The simultaneously obtained sample topography and optical amplitude $s_{n}$ images recorded at the *n*th demodulation order are shown in Figure 2C (optical amplitudes are normalized to the Au patch center). The topography shows a homogeneous Au patch, which is barely visible in the amplitude $s_{1}$ image, as the additive background is not suppressed. For higher *n*, the additive background is suppressed, allowing for a clearer recognition of the Au patch (compare $s_{1}$ to $s_{5}$ ). However, even at *n* = 5 the amplitude signal, *s*_5_, is not homogeneous (see also line profile Figure 2D), although it is expected for a homogeneous Au patch with homogeneous permittivity ϵ (yielding a constant $\alpha_{\text{eff,}5}^{\text{Au}}$ ).

To explain the variation of the amplitude signal $s_{5}$ on the Au patch (Figure 2D), we recall Eq. (2), where the far-field term ${\left(1+cr\right)}^{2}$ accounts for reflection of incident and tip-scattered field at the sample surface in front of the tip (illustrated by blue arrows in Figure 2B). Due to the diffraction-limited size of the far-field focus, the reflection occurs partially on Au and partially on ${\text{CaF}}_{2}$ (analogous to Figure 1C, panel (*ii*)), which we describe by

(4) $$cr=c_{\text{Au}}r_{\text{Au}}+c_{{\text{CaF}}_{2}}r_{{\text{CaF}}_{2}}$$

where $r_{\text{Au}}$ = 1 and $r_{{\text{CaF}}_{2}}$ = −0.06 are the far-field reflection coefficients [63, 64] of Au and CaF_2_. The weights $c_{\text{Au}}$ and $c_{{\text{CaF}}_{2}}$ depend on the respective areas of Au and ${\text{CaF}}_{2}$ that are illuminated by the incident light. When the sample is scanned below the tip (note that the tip position and laser focus are fixed), the weights $c_{i}$ and subsequently the reflection coefficient *r* are continuously changed, yielding a spatial variation of the demodulated and normalized near-field signal $\sigma_{n}$ , even when $\alpha_{\text{eff}}\left(\beta \right)$ is constant (as expected for the Au patch). Specifically, we explain the signal increase observed on Au from position 12–40 µm (Figure 2D) by an increase of *r*, as the illuminated area of Au increases and the illuminated area of ${\text{CaF}}_{2}$ decreases (compare schematics (*i*) and (*ii*) in Figure 2B). Analogously, we explain the signal decrease observed on ${\text{CaF}}_{2}$ from position 63–70 µm (Figure 2D zoom-in), by a decrease of *r*, as the illuminated area of Au decreases and the illuminated area of ${\text{CaF}}_{2}$ increases. We speculate that the s-SNOM signal is further modified by light scattered at the Au edge and thus additionally illuminates the tip [65] (illustrated by black arcs in Figure 2B), giving rise to rather complicated illumination (interference) artefacts near the edges of the Au patch.

**Figure 3:**
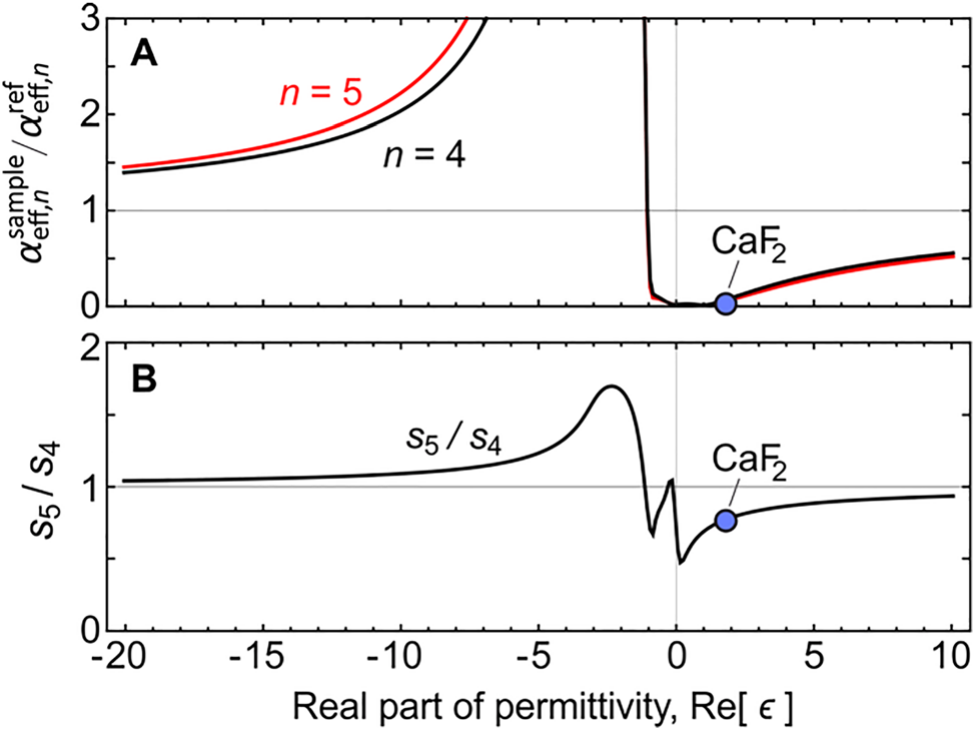
Calculated normalized s-SNOM signals. (A) Normalized *n*th Fourier coefficients of the effective polarizability, $\alpha_{\text{eff},n}^{\text{sample}}/\alpha_{\text{eff},n}^{\text{ref}}$ , as a function of Re[ ϵ ], where ϵ is the complex-valued dielectric function of the sample with Im[ ϵ ] = 0.2. As reference material we have chosen Au. (B) Amplitude ratio $s_{5}/s_{4}$ obtained from data of panel (A). Calculated using the finite dipole model for a tapping amplitude *A* = 75 nm and tip radius *R* = 30 nm (see Methods section). For comparison, blue symbols show experimental data obtained from Figure 2.

Interestingly, $\alpha_{\text{eff},n}^{\text{sample}}/\alpha_{\text{eff},n}^{\text{ref}}$ (describing the local near-field interaction between tip and sample) depends on the demodulation order *n* [1, 40] (Figure 3A), whereas the far-field term ${\left(1+cr\right)}^{2}$ in Eq. (3) is independent of *n* when it does not change nonlinearly with the tip-sample distance *z* on the scale of the tapping amplitude *A*. The latter condition is fulfilled when far-field reflection occurs at homogeneous sample areas that are large compared to the light wavelength *λ* and when A≪λ . In this case, the electric field distribution above the sample is governed by the interference of an incident and a reflected plane wave, yielding a sinusoidal variation of the total electric field as a function of *z* on a scale of *λ*. A tip that is oscillating with an amplitude *A* thus experiences a nearly constant far-field illumination within its whole oscillation cycle. Consequentially, the far-field reflection artefacts can be eliminated when the complex-valued ratio of $\sigma_{m}$ and $\sigma_{n}$ is calculated [60, 66], yielding a near-field signal $\sigma_{m}/\sigma_{n}$ that exclusively depends on the local near-field response:

(5) $$\frac{\sigma_{m}}{\sigma_{n}}=\frac{s_{m}}{s_{n}}{\text{e}}^{\text{i}\left(\varphi_{m}-\varphi_{n}\right)}=\frac{\alpha_{\text{eff},m}^{\text{sample}}/\alpha_{\text{eff},m}^{\text{ref}}}{\alpha_{\text{eff},n}^{\text{sample}}/\alpha_{\text{eff},n}^{\text{ref}}}\text{ with }m\neq n\text{.}$$

In Figure 3B we plot the calculated near-field amplitude ratio $s_{5}/s_{4}$ for a bulk sample of permittivity ϵ , normalized to an Au bulk sample. We find the same qualitative behavior as for $\alpha_{\text{eff},n}^{\text{sample}}/\alpha_{\text{eff},n}^{\text{ref}}$ (Figure 3A), although the contrast to Au (marked by horizontal line in Figure 3A and B), the contrast between different ϵ , and the spectroscopic contrast (see below) are reduced.

Applying Eq. (5) to the near-field images shown in Figure 2C, we find that the Au patch appears brighter than the ${\text{CaF}}_{2}$ substrate (Figure 2E, showing $s_{5}/s_{4}$ ), thus exhibiting a clear material (dielectric) contrast as predicted by the calculation shown in Figure 3B. Most importantly, the signal on Au is homogeneous, as expected for an Au film that has a homogeneous permittivity ϵ . Further, a good quantitative agreement between the experimental and calculated material contrast of ${\text{CaF}}_{2}$ (relative to Au) is found (see blue symbol in Figure 3B). We note that the amplitude ratio signal, $s_{5}/s_{4}$ , on ${\text{CaF}}_{2}$ is rather noisy, which can be explained by the very small amplitude signals $s_{5}$ and $s_{4}$ of ${\text{CaF}}_{2}$ (owing to its rather small permittivity $\epsilon_{{\text{CaF}}_{2}}=1.8$ ). We expect better signal-to-noise ratios for samples with larger permittivity ϵ (as confirmed by the experiments below). The comparison of Figure 2C and E clearly corroborates that the variations of the near-field amplitude $s_{n}$ on the Au patch (Figure 2D) are caused by far-field reflection of the incident and tip-scattered light at the sample surface, which are not suppressed in demodulated s-SNOM signals $\sigma_{n}$ but which can be suppressed when the ratio of two demodulated signals is calculated according to Eq. (5).

We note that the s-SNOM amplitude signals $s_{3}$ to $s_{5}$ are slightly reduced near the gold edges and on some spot-like areas on the gold surface. These darker regions correlate with elevated regions of the sample (the topography images in Figure 2C reveals small rims at the gold edges and small particles/grains on the gold surface, respectively, of about 10–15 nm height). The reduced amplitude signals can be thus explained by a reduced near-field interaction between the tip and the extended gold surface [67, 68], as the distance between them is increased. Being a near-field interaction effect rather than a far-field illumination effect, it is not eliminated when the ratio $s_{5}/s_{4}$ is calculated.

**Figure 4:**
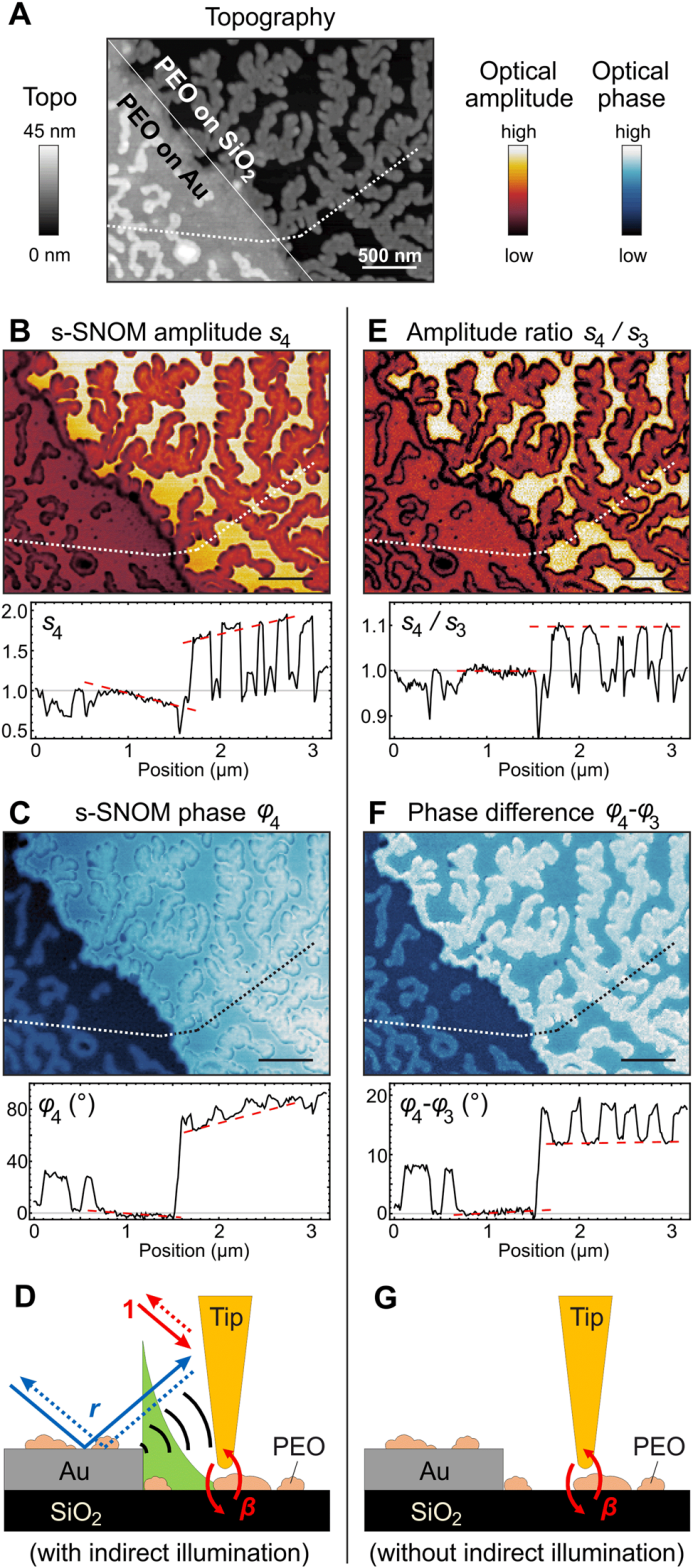
Elimination of material contrast variations induced by tip illumination via the sample surface. (A) Topography, (B) s-SNOM amplitude $s_{4}$ , (C) s-SNOM phase $\varphi_{4}$ , (E) amplitude ratio $s_{4}/s_{3}$ and (F) phase difference $\varphi_{4}-\varphi_{3}$ images and line profiles (taken along the dotted line in the images) of self-assembled PEO on Au and SiO_2_ surfaces, recorded with tapping amplitude *A* = 37 nm, tip radius *R* = 25 nm (PtIr tip, Nanoworld Arrow-NCPt) and illumination wavenumber $\nu =1114{\text{cm}}^{-1}$ . Optical signals are normalized to that of Au. Red dashed lines in the line profiles indicate the near-field signals on Au and SiO_2_. (D and G) Illustration of contributions to $\sigma_{n}$ in s-SNOM (D) and to the ratio $\sigma_{m}/\sigma_{n}$ (G), comprising direct beams (“1”), indirect beams caused by far-field reflections (“*r*”), and near-field reflections (“ β ”). Scale bars 500 nm.

In Figure 4, we exemplarily demonstrate with a more complex sample that far-field reflection and far-field scattering at the sample surface can prevent a reproducible and reliable quantitative determination of local dielectric contrasts, which, however, can be tackled by analyzing s-SNOM ratio images. To this end, we show the topography and s-SNOM images of 13 nm-thick poly(ethylene oxide) (PEO) islands on a partially Au covered quartz $\left({\text{SiO}}_{2}\right)$ surface (Figure 4A–C). We find again that the s-SNOM signals exhibit artificial gradients (indicated by red dashed lines in line profiles Figure 4B and C), although the permittivity of each material is expected to be homogeneous and the s-SNOM signals are free of additive and multiplicative background. We attribute the signal inhomogeneities to variations of (*i*) the illumination of the tip by light scattered at the Au edge (illustrated by black arcs in Figure 4D) and by propagating surface-phonon polaritons on ${\text{SiO}}_{2}$ (launched by the Au edge [63] and illustrated by the green drawing in Figure 4D that represents an evanescent wave) and (*ii*) the far-field reflection of the incident and tip-scattered light at the sample surface (illustrated by blue arrows in Figure 4D) when the sample is scanned. To quantify the material contrast variations, we evaluated the s-SNOM amplitude and phase contrasts between PEO and Au and between ${\text{SiO}}_{2}$ and Au by normalizing the near-field signals of different locations of PEO and ${\text{SiO}}_{2}$ to the near-field signals of different locations of Au (Supplementary Figure S1). We find that the amplitude contrasts $s_{4}^{\text{PEO}}/s_{4}^{\text{Au}}$ and $s_{4}^{{\text{SiO}}_{2}}/s_{4}^{\text{Au}}$ can vary by up to ±20% compared to the respective mean value. For the phase contrasts $\varphi_{4}^{\text{PEO}}-\varphi_{4}^{\text{Au}}$ and $\varphi_{4}^{{\text{SiO}}_{2}}-\varphi_{4}^{\text{Au}}$ we find variations of up to ±15%. As before in Figure 2, the calculated s-SNOM ratio images, $\sigma_{4}/\sigma_{3}$ , are much clearer and the material contrasts are more reproducible, i.e., we observe constant near-field signals on each material without artificial gradients (indicated by horizontal red dashed lines in line profiles of Figure 4E and F). Repeating the quantification of material contrasts (Supplementary Figure S1), we find significantly reduced variations of less than ±5% for the amplitude contrasts $\left[s_{4}^{{\text{SiO}}_{2}}/s_{4}^{\text{Au}}\right]/\left[s_{3}^{{\text{SiO}}_{2}}/s_{3}^{\text{Au}}\right]$ and a two-times reduced variation of the phase contrasts $\left[\varphi_{4}^{\text{PEO}}-\varphi_{4}^{\text{Au}}\right]-\left[\varphi_{3}^{\text{PEO}}-\varphi_{3}^{\text{Au}}\right]\text{.}$ These results highlight again the presence of signal variations on homogeneous materials in background-free s-SNOM amplitude $s_{n}$ and $\varphi_{n}$ images, which can be eliminated by calculating $s_{n+1}/s_{n}$ and $\varphi_{n+1}-\varphi_{n}$ images (illustrated in Figure 4D and G). We chose the demodulation order to be smaller in the denominator, since in this case the ratio $\sigma_{n+1}/\sigma_{n}$ exhibits the same qualitative behavior as $\sigma_{n}$ (see Figure 3).

### 2.3 Demonstration and elimination of far-field reflection artefacts in nano-FTIR spectroscopy

**Figure 5:**
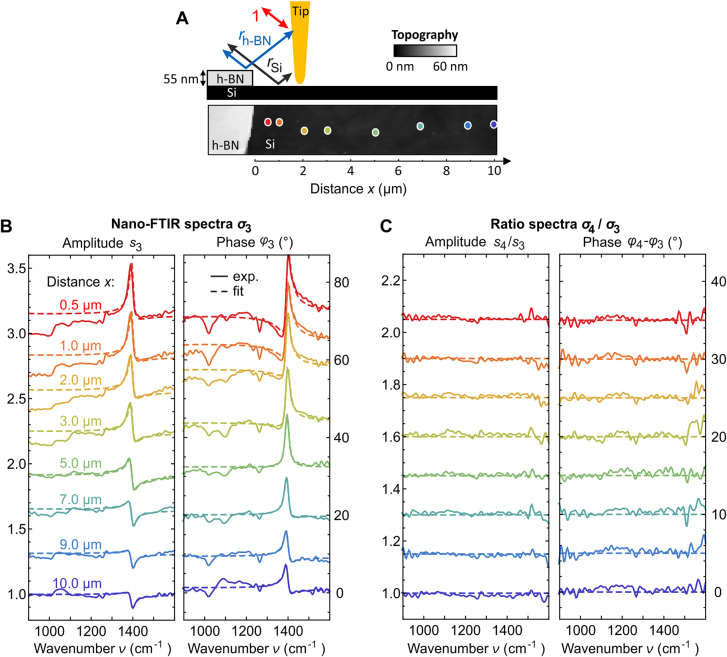
Analysis and removal of far-field reflection artefacts in nano-FTIR spectra. (A) Sketch of the experiment: nano-FTIR spectra are recorded on a Si substrate at various distances *x* between the tip and a 55 nm-thick h-BN flake, as indicated by colored dots in the topography image (bottom). The tip is illuminated directly (red arrow, “1”) and indirectly via h-BN (blue arrow, “ $r_{\text{h}-\text{BN}}$ ”) and Si (black arrow, “ $r_{\text{Si}}$ ”). (B) Experimental amplitude $s_{3}$ and phase $\varphi_{3}$ spectra (solid lines, normalized to the nano-FTIR spectrum of a Si reference sample) and results obtained by fitting a Fresnel reflection term as described in the main text to the spectral range 1300 cm^−1^–1500 cm^−1^ (dashed lines). Phase spectra $\varphi_{3}$ are baseline-corrected (see Methods section). (C) Amplitude ratio spectra $s_{4}/s_{3}$ and phase difference spectra $\varphi_{4}-\varphi_{3}$ corresponding to panel (B). Experiments were performed with tapping amplitude *A* = 30 nm, tip radius *R* = 25 nm (PtIr tip, Nanoworld Arrow-NCPt).

For a more detailed evaluation of the far-field reflection induced artefacts, particularly in nano-FTIR spectroscopy, we placed a monoisotopic hexagonal boron nitride [69] (h-BN) flake (of well-defined thickness and spectral response) onto a Si substrate (which is free of spectral features in the mid-infrared spectral range, $\epsilon_{\text{Si}}\approx 12$ ) [64]. We recorded spectra at various distances *x* relative to the h-BN flake, which is located in front of the tip (relative to the illumination direction, Figure 5A). The spectra are normalized to that of a clean Si reference substrate. We clearly observe a spectral feature at 1400 cm^−1^ in amplitude and phase, which decreases in magnitude and changes its spectral shape for increasing distances *x* up to 10 µm (Figure 5B). The spectral peak position matches that of the transverse optical (TO) frequency of h-BN [69], thus indicating that it is caused by far-field reflection at the h-BN flake (blue arrow in Figure 5A).

To verify and better understand the spectra shown in Figure 5B, we fit them with a model for the scattering coefficient $\sigma_{n}$ based on Eq. (3), considering that the sample spectra recorded on Si near the h-BN flake, $\sigma_{n}^{\text{sample}}\text{,}$ are normalized to a reference spectrum on Si recorded far away from h-BN, $\sigma_{n}^{\text{ref}}$ :

(6) $$\sigma_{n}=\frac{\sigma_{n}^{\text{sample}}}{\sigma_{n}^{\text{ref}}}=\frac{{\left[1+r\left(x\right)\right]}^{2}\alpha_{\text{eff},n}^{\text{Si}}}{{\left[1+cr_{\text{Si}}\right]}^{2}\alpha_{\text{eff},n}^{\text{Si}}}+b\text{.}$$

The weighted reflection coefficient *r*(*x*) of the sample accounts for far-field reflections that occur partially at the h-BN layer on Si (indicated by the blue arrow in Figure 5A; reflection coefficient $r_{\text{h}-\text{BN}}$ is calculated [46] from literature dielectric function [69] of h-BN) and partially at the bare Si surface (indicated by the black arrow in Figure 5A; reflection coefficient $r_{\text{Si}}$ is calculated [70] from literature dielectric function [64] of Si):

(7) $$r\left(x\right)=c_{\text{h}-\text{BN}}\left(x\right)r_{\text{h}-\text{BN}}+c_{\text{Si}}\left(x\right)r_{\text{Si}}\text{.}$$

The complex-valued weights $c_{\text{h}-\text{BN}}\left(x\right)$ and $c_{\text{Si}}\left(x\right)$ are fit parameters that depend on the distance *x* between h-BN flake and tip, determining the relative contribution of the far-field reflections at h-BN and Si. The spectrally constant offset *b* is another fit parameter to increase the robustness of the fit. For further fitting details and fit parameters see Supplementary Figure S2.

The fit results (dashed curves in Figure 5B) show excellent agreement with the experimental nano-FTIR data in amplitude and phase and for all distances *x*, showing that the demodulated and normalized signal $\sigma_{n}$ probes the local sample permittivity ϵ (via near field interaction with the Si sample, $\alpha_{\text{eff},n}^{\text{Si}}$ ), as well as the distant h-BN optical phonon via the far-field reflection coefficient *r* in the ${\left(1+cr\right)}^{2}$ term that multiplies with $\alpha_{\text{eff},n}^{\text{Si}}$ . As *r* depends on multiple parameters (such as the tip length, focus size and variation of the permittivity within the focal spot, summarized by the weights *c*_*i*_) it is difficult to accurately include it within the data analysis process and thus represents a severe issue for the quantitative measurement of the local sample properties. As before demonstrated with s-SNOM images (Figures 2 and 4), we can solve this issue by calculating complex-valued signal ratios, $\sigma_{n+1}/\sigma_{n}$ , but now applied to nano-FTIR spectra. The ratio spectra $\sigma_{4}\left(\nu \right)/\sigma_{3}\left(\nu \right)$ indeed exhibit a spectrally flat response (as expected for Si), thus demonstrating the elimination of the far-field spectral feature caused by the distant h-BN flake.

**Figure 6:**
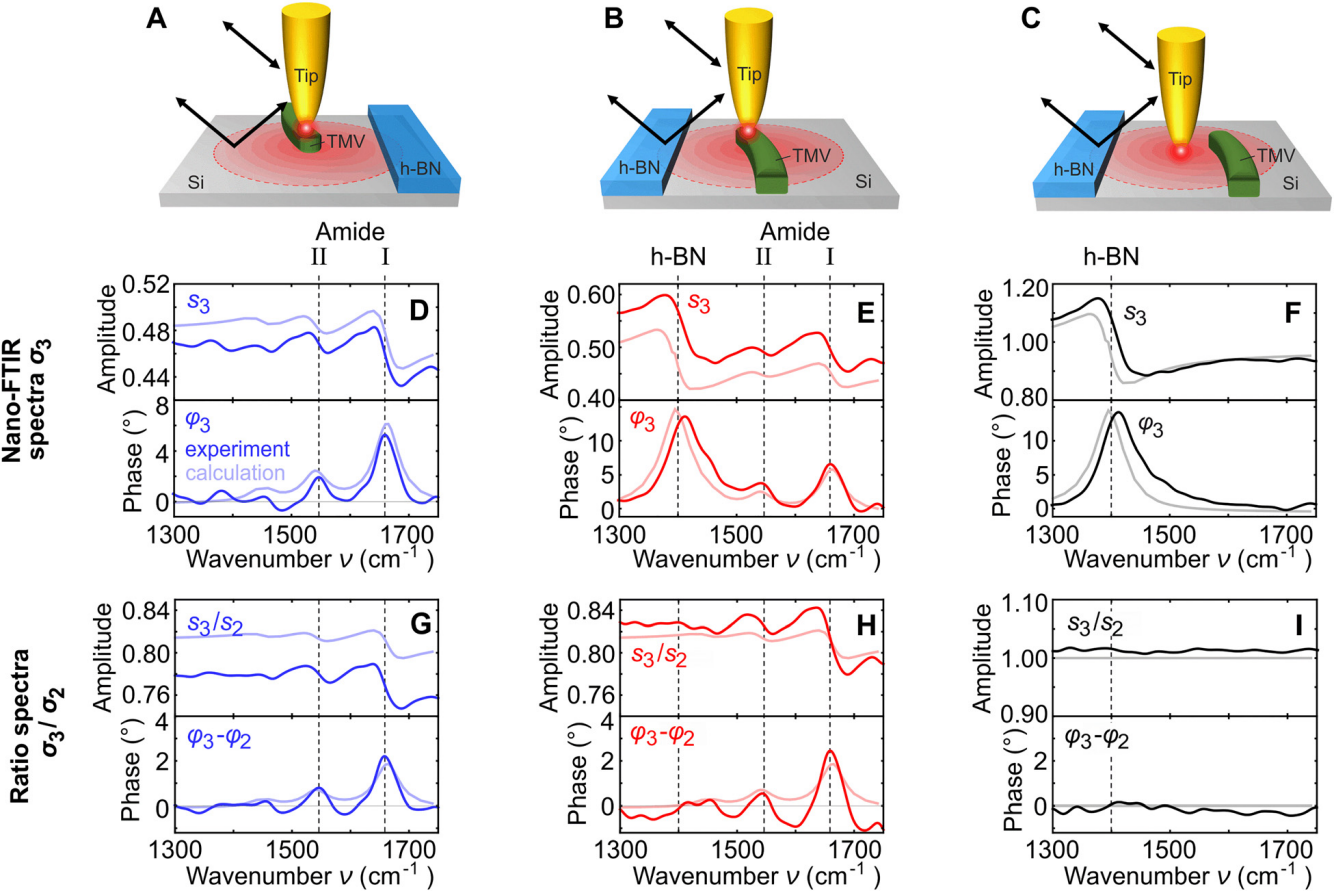
Comparison of experimental nano-FTIR spectra and their ratio of a single tobacco-mosaic-virus. (A–C) Illustration of nano-FTIR experiments with a TMV located at a distance of *x* ≈ 5 μm from a 150 nm-thick h-BN flake, both located on a Si substrate. (A) The tip is located on a TMV and illuminated directly and via reflection at the bare Si substrate, (B) same as panel (A) but the tip is illuminated via reflection at the h-BN flake, (C) the tip is located next to a TMV on Si and illuminated via reflection at the h-BN flake. (D–F) nano-FTIR amplitude $s_{3}$ (upper panels) and phase spectra $\varphi_{3}$ (lower panels) corresponding to panels A–C. Phase spectra $\varphi_{3}$ are baseline-corrected (see Methods section). (G–I) Amplitude ratio (upper panels) and phase difference spectra (lower panels), $s_{3}/s_{2}$ and $\varphi_{3}-\varphi_{2}$ . (D–I) All spectra were normalized to a reference nano-FTIR spectrum recorded on the Si substrate far away from TMV and h-BN. Vertical dashed lines at $\nu =1425{\text{cm}}^{-1}$ , $1545{\text{cm}}^{-1}$ and $1660{\text{cm}}^{-1}$ indicate the spectral positions of the h-BN TO phonon, amide II and amide I bands of the TMV, respectively. Dark colored curves show experimental spectra, light colored curves show calculated spectra. Experiments were performed using PtIr tips (Neaspec nano-FTIR probe) with an apex radius *R* = 40 nm and tapping amplitude *A* = 30 nm.

To mimic nano-FTIR spectroscopy of a nanoscale object in a crowded environment (analogous to Figure 1C, left panel), we studied a test sample comprising a single tobacco-mosaic-virus (TMV) next to a thin h-BN flake (both located on a large and clean Si substrate; sketched in Figure 6A). Measuring a nano-FTIR spectrum on the TMV without h-BN being located in front of the tip (A), we see exclusively the TMV’s characteristic amide I and amide II bands [33] (Figure 6D). However, by rotating the sample such that the tip is illuminated via reflection at the h-BN flake (Figure 6B), we see an additional and pronounced spectral peak at 1400 cm^−1^ (Figure 6E). As illustrated before in Figure 5, it can be associated with the TO phonon of h-BN. For verification we recorded another nano-FTIR spectrum at a distance of about 300 nm to the virus (Figure 6C). It indeed shows exclusively the peak at 1400 cm^−1^, but not amide I and II bands of the TMV (Figure 6F).

Figure 6 highlights the dramatic modification of the spectral signature of an isolated nanoscale object due to the presence of an adjacent large-scale structure. In the case that the environment of the object of interest (here the TMV) is not known or of a highly complex composition, such strong additional spectral features may lead to severe misinterpretations and wrong chemical identification of the object. We can solve this problem, as before in Figure 5, by analyzing the ratio $\sigma_{3}\left(\nu \right)/\sigma_{2}\left(\nu \right)$ . Figure 6G confirms that the ratio spectra reproduce well the amide I and II bands of TMV. More importantly, the ratio spectra do not reveal anymore the h-BN optical phonon (i.e., the spectral feature of the environment) when the tip is illuminated via reflection at the h-BN flake (Figure 6 H and I) but instead reveal exclusively the amide I and II bands of the TMV (Figure 6H).

The experimental results are qualitatively confirmed by calculations using the finite dipole model [61] (light colored curves in Figure 6D–I), where the TMV was assumed to be an infinitely extended layer [71] with a dielectric function described by three Lorentz-oscillators (manually adjusted to reproduce the experimentally observed amide I and II bands; calculation details in Methods section). Interestingly, the nano-FTIR spectrum $\sigma_{3}$ and the ratio $\sigma_{3}/\sigma_{2}$ of the TMV are essentially identical (Figure 6D, G and H), showing that the spectral ratio can be interpreted analogously to nano-FTIR spectra [15], while being free of artefacts caused by tip illumination via far-field reflection at the sample. We note that the spectral contrast is reduced in the ratio spectra (in the case of the TMV by approximately 50%), which, however, still allows for reliable analysis and chemical identification even for small nanoobjects such as a single TMV.

**Figure 7:**
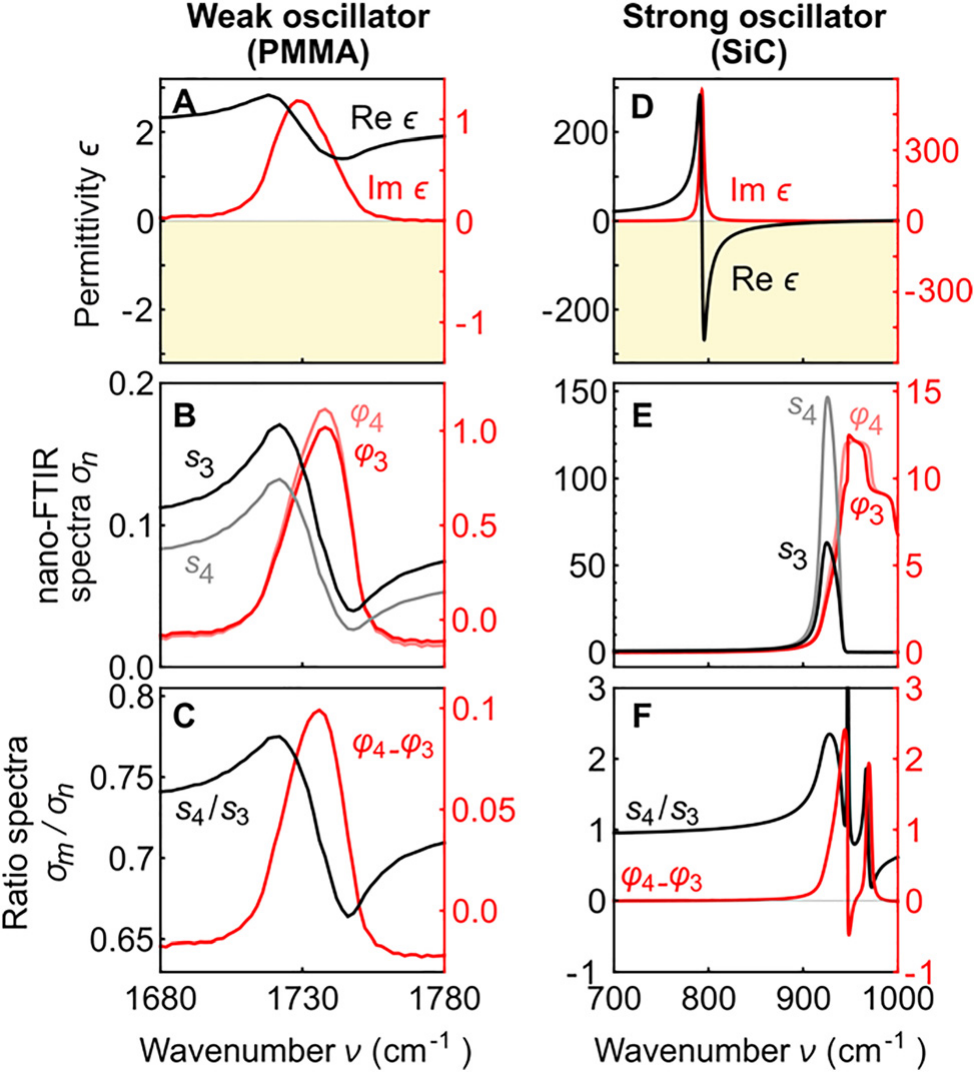
Comparison of calculated nano-FTIR spectra and their ratios for weak and strong oscillator samples. (A) Real and imaginary part of the dielectric function ϵ of a weak oscillator (C=O molecular vibrational mode of PMMA). (B) nano-FTIR amplitude and phase spectra $s_{n}$ and $\varphi_{n}$ of the weak oscillator at demodulation orders *n* = 3 and 4, calculated using the finite dipole model for a tapping amplitude *A* = 30 nm and tip radius *R* = 40 nm (see Methods section). (C) nano-FTIR amplitude ratio, $s_{4}/s_{3}$ , and phase difference, $\varphi_{4}-\varphi_{3}$ , spectra of the weak oscillator. (D–F) Analog to A–C, but for a strong oscillator (TO phonon of SiC).

For further insights, we compare in Figure 7 the dielectric function of the sample, ϵ , calculated nano-FTIR spectra $\sigma_{n}$ and ratio spectra $\sigma_{m}/\sigma_{n}$ of weak and strong oscillator samples. As representative sample materials we have chosen polymethyl-methacrylate [72] (PMMA, Figure 7A–C) and SiC [73] (Figure 7D–F), respectively. For the weak oscillator we find that the spectral contrast in the near-field amplitude $s_{n}$ and phase $\varphi_{n}$ spectra (difference between amplitude maximum and minimum and height of the phase peak, respectively) is increased at higher demodulation orders *n*, while the spectral peak (dip) positions are preserved (Figure 7B, spectra normalized to Au reference sample). Calculating the ratio $\sigma_{4}/\sigma_{3}$ thus yields amplitude $s_{4}/s_{3}$ , and phase $\varphi_{4}-\varphi_{3}$ spectra (Figure 7C) that qualitatively resemble the $s_{n}$ and $\varphi_{n}$ spectra, as well as the real and imaginary parts of the dielectric function ϵ [72]. This allows for a straight-forward chemical identification of weak oscillator materials via ratio spectra, particularly from the phase spectra that are known to match well the standard far-field absorption fingerprint of molecular vibrations [5, 15, 74].

For comparison, we show in Figure 7D the dielectric function for the strong oscillator sample, where the near-field amplitude and phase spectra (Figure 7E), $s_{n}$ and $\varphi_{n}\text{,}$ reveal a resonant tip-sample near-field interaction due to excitation of localized surface polaritons near ϵ≈−1 [11, 47, 75]. The near-field spectra thus do neither match qualitatively the dielectric function nor standard far-field spectra. On the other hand, we find large differences between the near-field spectra obtained at different demodulation orders. Consequently, the calculated ratio spectra yield strong spectral features (Figure 7F), which, however, do not match qualitatively the $s_{n}$ and $\varphi_{n}$ spectra, and thus are less straight-forward to interpret. We attribute the complicated spectral features in the ratio spectra to a strong frequency shift of the polariton resonance when the tip-sample distance is varied during the tip oscillation, yielding resonance peaks that shift with increasing demodulation order [76, 77]. Further studies, particularly experimentally, are needed to elucidate how well the ratio spectra are suited for extracting strong oscillator near-field spectra in the presence of non-negligible far-field reflection at the sample surface.

### 2.4 Discussion

We note that far-field reflection artefacts for specific samples can be avoided by orienting the sample such that the tip is illuminated exclusively via the substrate that is used as reference (illustrated in Figure 1B), as we show for the TMV sample discussed in Figure 6 (compare panels (A, D) with (C, F)) and for the h-BN sample discussed in Figure 5 in Supplementary Figure S3. This is because the far-field terms ${\left(1+cr\right)}^{2}$ cancel when the near-field signal, respectively, nano-FTIR spectrum, of the object is normalized to the substrate according to Eq. (3), as the far-field reflection coefficients for the measurement and reference positions are nearly identical. However, the objects of interests (i.e., measurement positions) in many samples can have a crowded and unknown environment, where the ${\left(1+cr\right)}^{2}$ term is different for measurement and reference positions. In this case, it is unavoidable that the unknown far-field reflection coefficient *r* enters the normalized and background-free near-field signal $\sigma_{n}$ , which could lead to a misinterpretation of the local material properties. The ratio $\sigma_{m>n}/\sigma_{n}$ is thus an essential means for extracting the true local sample response, although the signal-to-noise ratio and the dielectric near-field contrasts are reduced. In the future, more sophisticated signal processing involving various signal harmonics may help to overcome these drawbacks. We also foresee a quantitative reconstruction of the local dielectric function based on the ratio $\sigma_{m>n}/\sigma_{n}$ , analogous to the reconstruction of the local dielectric function from the near-field signals $\sigma_{n}$ [62, 78]. This will be particularly important for layered samples with layer thicknesses on the scale of the tip radius, where $\sigma_{m}/\sigma_{n}$ depends on the vertical structure of the sample [18], since the probing depth of s-SNOM and nano-FTIR depends on the demodulation order *n* [78–80].

In this work, we have chosen to calculate the ratio of near-field signals that were normalized to reference near-field signals, $\sigma_{m}/\sigma_{n}=\left[\sigma_{m}^{\text{sample}}/\sigma_{m}^{\text{ref}}\right]/\left[\sigma_{n}^{\text{sample}}/\sigma_{n}^{\text{ref}}\right]$ . Even the ratio $\sigma_{m}^{\text{sample}}/\sigma_{n}^{\text{sample}}$ may be applied to eliminate the far-field reflection artefacts, as the ratio of the reference signals in Eq. (5), $\sigma_{m}^{\text{ref}}/\sigma_{n}^{\text{ref}}$ , merely represent a sample-independent constant factor. This possibility is particularly beneficial when a sample lacks suitable reference areas.

We note that the ratio of near-field signals of different demodulation orders – independent of whether normalized or not normalized to a reference sample – also eliminates the instruments’ response function, interferometer phase fluctuations (e.g. due to drift of the reference mirror; see Methods) and fluctuations of the laser output spectrum (Supplementary Figure 1). It can be thus also applied to perform imaging and spectroscopy without the need of a reference area or a reference sample (as proposed in [66]), as well as for reducing noise caused by laser and interferometer instabilities.

In the future, ratio images may also help to eliminate far-field illumination contributions in s-SNOM applications that aim on mapping the electric near-field distribution of propagating waveguide modes, polaritons or antenna fields using metallic or dielectric probing tips [42], [43], [44], [45], [46], [47], [48], [49], [50], [51], [52], [53], [54], [55], [56], [57]. This application will require that the near fields of interest decay nonlinearly on the scale of the tapping amplitude *A*, such that the corresponding near-field signals depend on the demodulation order *n* and do not cancel when ratio images are calculated. We expect that this condition will be fulfilled for highly confined polariton modes [46], [47], [48], [49], [50], [51], for example, in 2D materials, for which the fields normal to the surface decay on the scale of 100 nm and thus on the scale of the tapping amplitude *A*. For weakly confined modes, such as surface polaritons on bulk materials, the calculation of ratio images may eliminate the modes’ field distribution together with the far-field reflection, which can be observed in Figure 4, where the inhomogeneous signal on the SiO_2_ surface – originating from surface phonon polaritons launched by the gold edge – is eliminated in the ratio images.

We finally note that in other scanning probe techniques employing diffraction-limited tip illumination, such as tip-enhanced photothermal infrared nanospectroscopy [81] and photoinduced force microscopy [82, 83], the tip is also illuminated indirectly via far-field reflection at the sample. We thus speculate that, similarly to s-SNOM and nano-FTIR, these techniques may also yield artificial signal gradients and (spectral) signatures of materials that are not present below the tip, which should be elucidated in future work.

## 3 Conclusions

In conclusion, we showed that unavoidable far-field reflections at the sample surface can potentially introduce quantitative and qualitative artefacts in s-SNOM and nano-FTIR spectroscopy, with the severity depending on the specific sample geometry. In many cases, these artefacts can be avoided or minimized, for example, by appropriate sample orientation or data analysis that includes the appropriate Fresnel reflection coefficient. However, for the case of complex sample geometries, where far-field reflections cannot be avoided or accounted for, the analysis of ratio images and ratio nano-FTIR spectra, $\sigma_{m>n}/\sigma_{n}$ , can eliminate the reflection artefacts. The power of this method is the exact cancellation of the far-field reflection, since all the data necessary is measured simultaneously on the same exact pixel. The reduced signal-to-noise ratio and material contrasts may be circumvented in the future by more sophisticated methodologies.

## 4 Methods

### 4.1 Finite dipole model

Nano-FTIR spectra were calculated using the finite dipole model (FDM) for semi-infinite samples [61] with an extension to layered samples [71]. The model describes the tip as an elongated spheroid (with major half-axis length *L* = 300 nm) that oscillates vertically above the sample. The empirical model parameter *g* = $0.7{\text{e}}^{0.06\text{i}}$ describes how much of the charge induced in the tip is relevant for near-field interaction [61]. In our simulation we used the tapping amplitude *A* = 30 nm for Figures 6 and 7, and *A* = 75 nm for Figure 3. We approximate the dielectric function of the TMV (Figure 6) by a sum of three Lorentz oscillators (to model the amide I, II and III bands; units given in cm^−1^ except $\epsilon_{\infty }$ which is unity-free): $\epsilon_{\text{TMV}}=\epsilon_{\infty }+\sum_{k=1}^{3}A_{k}^{2}/\left(\nu_{k}^{2}-\nu^{2}-i\nu \gamma_{k}\right)$ with $\epsilon_{\infty }=2$ , $A_{1}=150$ , $A_{2}=90$ , $A_{3}=60$ , $\nu_{1}=1660$ , $\nu_{2}=1540$ , $\nu_{3}=1450$ , $\gamma_{1}=50$ , $\gamma_{2}=50$ and $\gamma_{3}=50$ . For the Si substrate we use [64] $\epsilon_{\text{Si}}\approx 12$ .

### 4.2 Baseline-correction

In Figures 5 and 6 we plot baseline-corrected nano-FTIR phase spectra to remove two effects: (*i*) a tilted phase baseline, which is caused by thermal drift of the interferometer white-light-position (WLP) between and during the reference and sample measurements [17], and (*ii*) a small negative phase offset, observed on organic nanostructures [12]. To determine the tilt of the baseline caused by the WLP drift, we compare two reference measurements: one taken before and one taken after the sample measurements ( $\sigma_{\text{ref},n}^{\left(1\right)}$ and $\sigma_{\text{ref},n}^{\left(2\right)}$ , respectively). As the same material is probed, we expect the two reference signals to be exactly identical (within the noise limit). However, when the WLP drifts, we observe a tilted baseline when plotting $\sigma_{\text{ref},n}^{\left(1\right)}/\sigma_{\text{ref},n}^{\left(2\right)}=1{\text{e}}^{\text{i}2\pi \nu \cdot \Delta \text{WLP}}$ , where ΔWLP is the total distance that the WLP has moved during the total time $\Delta t_{\text{tot}}$ of the experiment (i.e., from the first reference measurement, over the sample measurements, to the second reference measurement). For a specific sample measurement *j* (recorded a time $\Delta t_{j}$ after the first reference measurement) the baseline tilt can be removed by calculating $\left(\sigma_{\text{sample},n}^{\left(j\right)}/\sigma_{\text{ref},n}^{\left(1\right)}\right)\cdot \text{exp}\left[-i2\pi \nu \Delta \text{WLP}\Delta t_{j}/\Delta t_{\text{tot}}\right]$ , assuming that the speed of the WLP drift is constant throughout the whole experiment (typically on the order of 50 nm per minute [17]). In a second step, we remove the negative phase offset by simply shifting the curve vertically, such that the phase is zero at frequencies where the samples are known to be nonabsorbing. Note that the WLP-drift is independent of the demodulation order *n*, and thus does not affect the ratio $\sigma_{m}/\sigma_{n}$ . In other words, calculating the ratio $\sigma_{m}/\sigma_{n}$ provides an elegant way to remove instabilities of the interferometer WLP.

## Supplementary Material

Supplementary Material
