# Supplementary material for: High-fidelity nano-FTIR spectroscopy by on-pixel normalization of signal harmonics
Source: Nanophotonics. 2021 Dec 13;11(2):377–90. doi: 10.1515/nanoph-2021-0565 (PMC11501567; doi:10.1515/nanoph-2021-0565)
Supplement: Supplementary file 1 — Supplementary Material [file j_nanoph-2021-0565_suppl.pdf]

Supplementary Material

High-fidelity nano-FTIR spectroscopy by on-pixel normalization of signal harmonics

Lars Mester[1], Alexander A. Govyadinov[2] and Rainer Hillenbrand*[3,4]

[1] CIC nanoGUNE BRTA, 20018 Donostia-San Sebastian, Spain
[2] neaspec GmbH, Eglfinger Weg 2, 85540, Munich-Haar, Germany
[3] CIC nanoGUNE BRTA and Department of Electricity and Electronics, EHU/UPV, 20018 Donostia-San Sebastian, Spain
[4] IKERBASQUE, Basque Foundation for Science, 48011 Bilbao, Spain

*Correspondence and material requests should be addressed to R.H.
(r.hillenbrand@nanogune.eu)

Table of contents

S1 – Comparison of material contrasts observed on the PEO on Au and SiO_2 sample

S2 – Fit parameters and description of fitting procedure regarding Figure 5

S3 – Avoiding far-field reflection artefacts by sample rotation

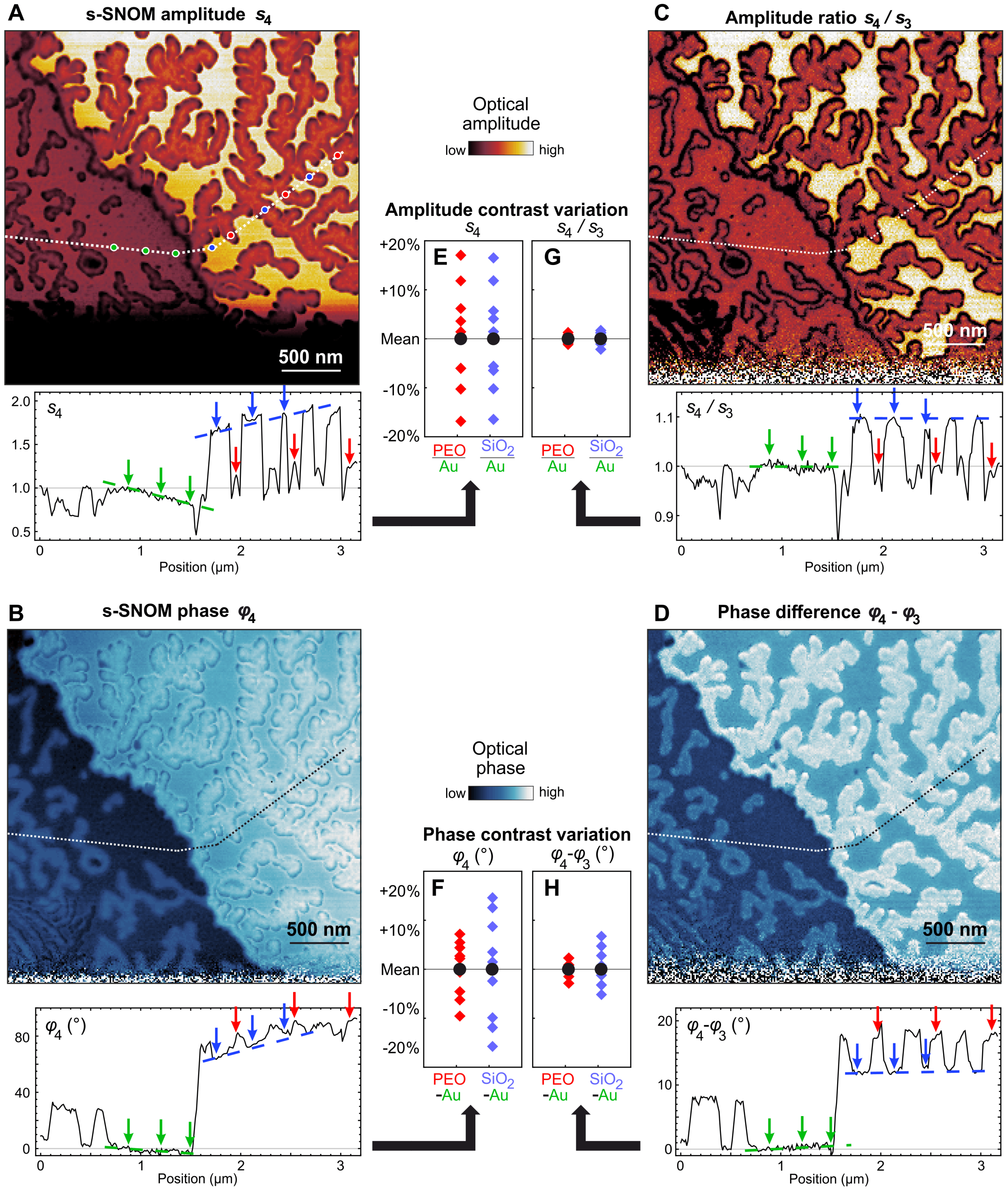

Supplementary Figure S1: Comparison of material contrasts observed on the PEO on Au and SiO_2 sample. (A) s-SNOM amplitude s_4, (B) s-SNOM phase φ_4, (C) amplitude ratio s_4/s_3 and (D) phase difference φ_4-φ_3 images and line profiles (taken along the dotted line in the images) of self-assembled PEO on Au and SiO_2 surfaces. Same data as Fig. 4, but showing more lines at the bottom of the images. (E,F) Amplitude and phase contrasts s_4^{PEO}/s_4^{Au}, s_4^{SiO2}/s_4^{Au}, $\varphi_4^{PEO} - \varphi_4^{Au}$ and $\varphi_4^{SiO2} - \varphi_4^{Au}$, obtained by normalizing different locations on PEO (marked by red arrows) and SiO_2 (blue arrows) to different locations on Au (green arrows). Contrasts are shown relative to their respective mean values. (G,H) Amplitude ratio and phase difference contrasts, analogous to (E,F). Note that the s-SNOM amplitude s_4 rapidly vanishes at the bottom of the image (panel A), which we attribute to a decrease in the illuminating laser power, caused by laser instability. Interestingly, the amplitude ratio s_4/s_3 is not affected until the noise becomes larger than the observed dielectric contrasts, showing that changes in the tip illumination are compensated by calculating s_4/s_3.

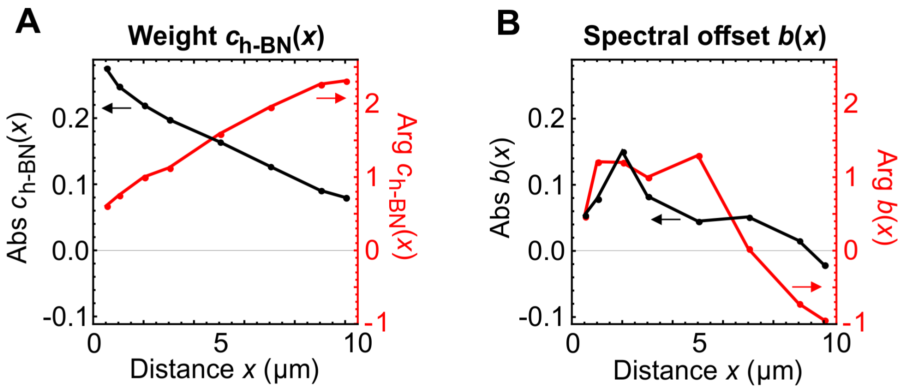

Supplementary Figure S2: Fit parameters and description of fitting procedure regarding Figure 5. (A) Weight $c_{\text{h-BN}}(x)$ and (B) spectral offset $b(x)$ obtained by fitting equation (6) and (7) to the experimental data in Fig. 5B (spectral range 1300 cm^{-1} to 1500 cm^{-1}), assuming an angle of incidence $\Theta = 60°$ with respect to the sample surface normal. There are three different weights: (*i*) The weight c (without index) generally describes the weight of the reflected beam compared to the direct illumination, when a homogeneous material is placed in front of the tip (as for the reference measurement on silicon). (*ii*) The weight $c_{\text{h-BN}}(x)$ and (*iii*) weight $c_{\text{Si}}(x)$ describe the distance-dependent weight of the beams reflected at h-BN, respectively Si, compared to the direct illumination. We assume for all distances x that the sum of weights is constant: $c_{\text{h-BN}}(x) + c_{\text{Si}}(x) = c$. For fitting the nano-FTIR spectrum that is closest to the h-BN flake (x = 500 nm), we assume that far-field reflections take place only at the h-BN surface (neglecting reflections at the 500 nm-wide silicon area). We obtain $c \approx c_{\text{h-BN}}(0)$. For fitting the nano-FTIR spectra at all other distances x, we keep c constant in the denominator of equation (6) and apply $c_{\text{Si}}(x) = c - c_{\text{h-BN}}(x)$ in equation (7), such that the only free fit parameters are $c_{\text{h-BN}}(x)$ and $b(x)$. Note that the weight c was introduced in an earlier work [1] but was neither quantified nor analysed. According to our analysis we find $c = 0.28e^{i0.6}$. The fitting results further show that Abs[$c_{\text{h-BN}}(x)$] decreases and Arg[$c_{\text{h-BN}}(x)$] increases (both nearly linearly) for increasing distance x between tip and h-BN. The decrease of Abs[$c_{\text{h-BN}}(x)$] can be explained by the reduced amount of light reflected at the h-BN flake that is illuminating the tip. The increase of Arg[$c_{\text{h-BN}}(x)$] we explain by the increasing distance between the h-BN flake to the tip. Altogether, the varying nano-FTIR peak shapes observed in Fig. 5B result from the varying interference between the direct and reflected fields that illuminate the tip, the specific peak shapes being governed by the relative weights of the two fields and the phase shift between them.

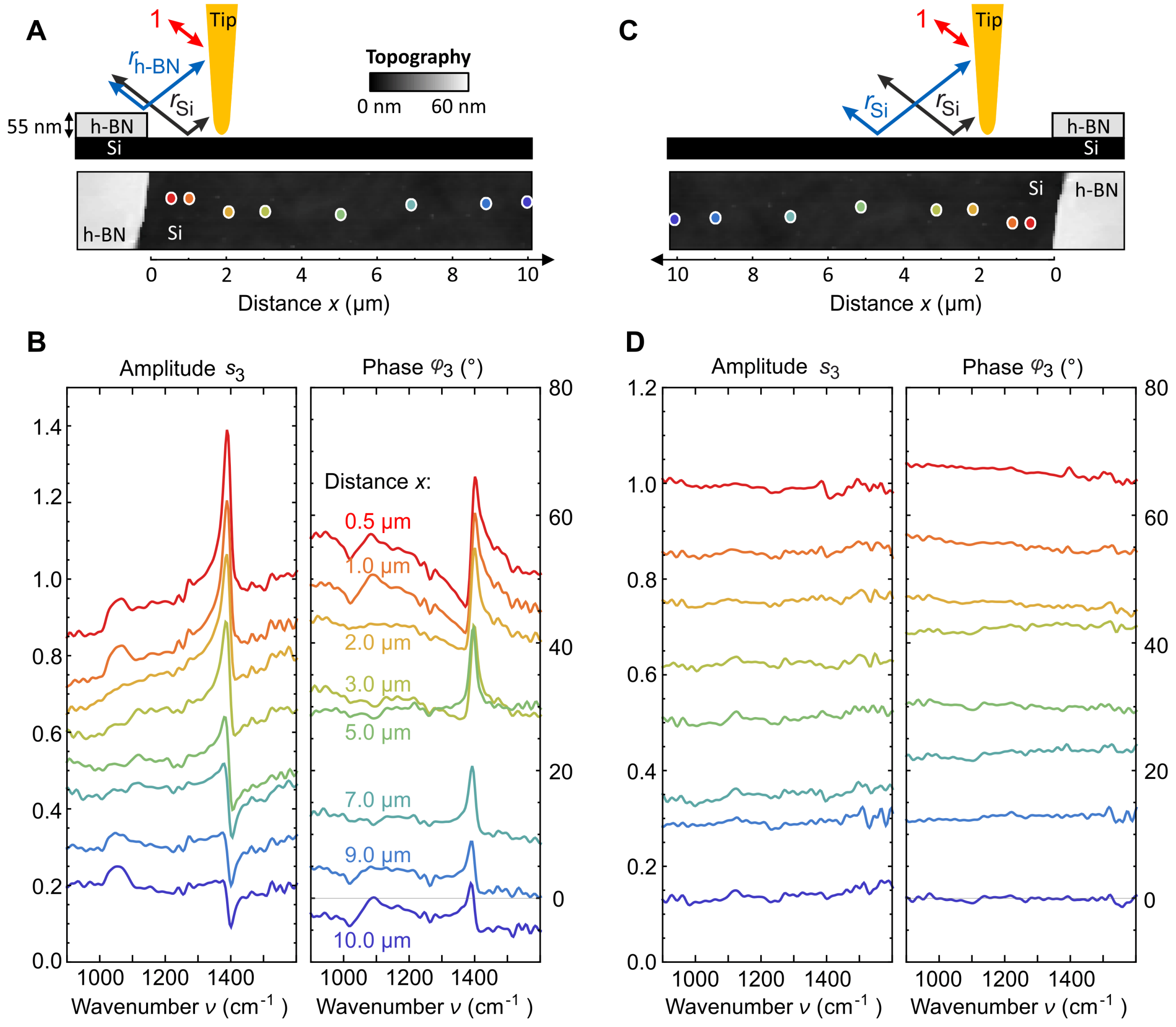

Supplementary Figure S3: Avoiding far-field reflection artefacts by sample rotation. (A) Sketch of experiment and topography image: same as Fig. 5A. (B) Experimental amplitude s_3 and phase φ_3 spectra (normalized to the nano-FTIR spectrum of a Si reference sample). (C,D) Sketch and experimental data analogous to panels a and b, but the sample is rotated by 180° such that the tip is illuminated directly and via reflection exclusively at the Si substrate. The figure shows that for this specific sample the far-field reflection artefacts can be avoided simply by sample rotation, as discussed in the main text.

REFERENCES

[1] J. Aizpurua, T. Taubner, F. Javier Garcia de Abajo, M. Brehm, and R. Hillenbrand, “Substrate-enhanced infrared near-field spectroscopy,” *Optics Express*, vol. 16, no. 3, pp. 1529–1545, Feb. 2008, doi: 10.1364/OE.16.001529.
